# Action upon the Fœtus of Medicine Taken by the Mother

**Published:** 1881-12

**Authors:** 


### ACTION UPON THE FŒTUS OF MEDICINE TAKEN BY THE MOTHER.

Using the microphone, Dr. Kubassow has observed the effect on the heart of the fœtus of medicine taken by the mother. (*Dissertation: Russisch. Centralb. fur Gynækol.*) 1. Chloroform and chloral-hydrate, he finds, have first a stimulant, then a sedative effect on the fœtus, this last effect being evidenced by the dullness and the infrequency of the heart's beat, and the greater quietness of the fœtus. They act within five to ten minutes, chloral more powerfully than chloroform, and especially so if given per rectum. 2. Opium and alkaloids cause prolonged irregularity of the heart's beat in the fœtus, acting more slowly, but for a longer period than chloral or chloroform. Opium acts more powerfully per os than per rectum. 3. Digitalis has also a powerful and prolonged action on the fœtus. Dr. Kubassow believes, from chemical examination, that a dose of chloral hydrate taken by the mother is divided within fifteen minutes between herself and her child, in proportion to their weights. The practical conclusion is, that more than thirty grains of chloral hydrate will be dangerous to the child, if given at once per rectum, or repeated sooner than in half an hour. The same holds for one and a half grains of opium, as tincture, repeated sooner than in an hour.--*Medical Press and Circular.*

The true function of a medical society is to gather together and then diffuse knowledge, to encourage independent inquiry, to survey from time to time, by the light of mutual reflection, the positions attained, and thus to seek sound guidance in the application of our knowledge to our practical duties.—*Robert Barnes.*

Dr. D. J. Snyder, of Scio, O., writes that he will furnish the seeds of the eucalyptus globulus, with directions to plant, to any medical gentleman who desires to attempt the effort to raise them. He is sure that if the plants be kept for the first winter in a hot-house, they would gain strength sufficient to enable them to endure the following winters.

